# The Genetic and Epigenetic Footprint in Idiopathic Pulmonary Fibrosis and Familial Pulmonary Fibrosis: A State-of-the-Art Review

**DOI:** 10.3390/diagnostics12123107

**Published:** 2022-12-09

**Authors:** Claudio Tirelli, Chiara Pesenti, Monica Miozzo, Michele Mondoni, Laura Fontana, Stefano Centanni

**Affiliations:** 1 Respiratory Unit, ASST Santi Paolo e Carlo, Department of Health Sciences, University of Milan, 20142 Milan, Italy; 2 Medical Genetics Unit, ASST Santi Paolo e Carlo, Department of Health Sciences, University of Milan, 20142 Milan, Italy

**Keywords:** idiopathic pulmonary fibrosis, familial pulmonary fibrosis, genetic, epigenetic, MUC5B, telomere, telomerase, surfactant, ABCA3, therapy

## Abstract

Idiopathic pulmonary fibrosis (IPF) is a rare disease of the lung with a largely unknown etiology and a poor prognosis. Intriguingly, forms of familial pulmonary fibrosis (FPF) have long been known and linked to specific genetic mutations. There is little evidence of the possible role of genetics in the etiology of sporadic IPF. We carried out a non-systematic, narrative literature review aimed at describing the main known genetic and epigenetic mechanisms that are involved in the pathogenesis and prognosis of IPF and FPF. In this review, we highlighted the mutations in classical genes associated with FPF, including those encoding for telomerases (*TERT*, *TERC*, *PARN*, *RTEL1*), which are also found in about 10–20% of cases of sporadic IPF. In addition to the Mendelian forms, mutations in the genes encoding for the surfactant proteins (*SFTPC*, *SFTPA1*, *SFTPA2*, *ABCA3*) and polymorphisms of genes for the mucin *MUC5B* and the Toll-interacting protein TOLLIP are other pathways favoring the fibrogenesis that have been thoroughly explored. Moreover, great attention has been paid to the main epigenetic alterations (DNA methylation, histone modification and non-coding RNA gene silencing) that are emerging to play a role in fibrogenesis. Finally, a gaze on the shared mechanisms between cancer and fibrogenesis, and future perspectives on the genetics of pulmonary fibrosis have been analyzed.

## 1. Introduction

Idiopathic pulmonary fibrosis (IPF) is a rare, chronic, fibrosing and progressive disease limited to the lung with a largely unknown etiology and a poor prognosis. IPF is associated with a characteristically defined radiological and/or histological usual interstitial pneumonia (UIP) pattern. IPF is characterized by the progressive replacement of lung tissue with fibrotic-scar tissue due to the excessive release of collagen by mesenchymal cells, the myofibroblasts. This process changes the architecture and function of the organ over time and, in the absence of antifibrotic therapy, may lead to death within 3–5 years after diagnosis. IPF is a disease of the elderly, with a mean age of onset of 65 years [1].

IPF is characterized by the presence of progressively worsening dyspnea. Cough and sputum are frequently associated symptoms, especially in more severe patients.

According to international guidelines, the diagnosis requires the exclusion of secondary causes of pulmonary fibrosis (i.e., connective tissue diseases) and the execution of a high-resolution computed tomography (HRCT) of the chest. Finding a definite usual interstitial pneumonia (UIP) radiological pattern is sufficient to diagnose IPF after the exclusion of any other causes of lung disease. Patients who have a probable UIP pattern on HRCT can receive a diagnosis of IPF after a multidisciplinary discussion (MDD) by at least a pulmonologist, a radiologist and a pathologist. Lung biopsy is today adopted only in limited complex cases, particularly when an IPF diagnosis is strongly suspected but the radiological pattern is indeterminate for UIP or suggestive for other interstitial lung diseases.

Bronchoalveolar lavage (BAL) is a bronchoscopic sampling procedure useful in the diagnostic work-up of idiopathic pulmonary fibrosis that helps rule out other fibrosing interstitial diseases that can enter into differential diagnosis with IPF. Although there are no specific cellular patterns for IPF, BAL could help in the diagnosis of other interstitial lung diseases (ILD) such as sarcoidosis, eosinophilic pneumonia, chronic extrinsic allergic alveolitis or nonspecific interstitial pneumonitis [2,3].

To date, no pharmacological therapy can stop or reverse the fibrosing process. Two drugs, nintedanib and pirfenidone, have been approved for the treatment of the disease, since in clinical trials and observational studies they have been shown to slow down the progression of the disease, without, however, being able to stop or cure it [4,5]. Several clinical trials are underway to expand the spectrum of antifibrotic drugs, but, to date, the only curative intervention remains lung transplantation, a therapeutic option applicable to a very small group of IPF patients respecting stringent selection criteria and with numerous possible complications.

Despite several advances in understanding the pathogenetic mechanisms of the disease, the origin of IPF remains largely unknown. The main risk factors include cigarette smoking, lung infections (especially viral, and, to a lesser extent, bacterial infections), exposure to pneumotoxic inhalants, gastroesophageal reflux, elderly age, diabetes mellitus and genetic predisposition [6].

Intriguingly, forms of familial pulmonary fibrosis (FPF) have long been known and linked to mutations in specific genes (Table 1). FPF is defined when the disease is diagnosed in the same family with at least 2 relatives developing pulmonary fibrosis. Altogether, these forms represent up to 20% of cases of pulmonary fibrosis [7].

Genes associated with FPF include those encoding for telomerases, responsible for the regulation of telomeres length and therefore involved in cellular aging (*TERT*, *TERC*, *PARN*, *RTEL1*) [8,9,10]. Two of the telomerase-related forms can also be characterized by bone marrow failure (PFBMFT1 and PFBMFT2 due to *TERC* and *TERT* mutations, respectively). The involvement of bone marrow function in the pulmonary fibrosis (PF) phenotype is also reported for patients with *ZCCHC8* and *RPA1* variants. The familiar forms of PF include ILD2 (OMIM #178500), a disease related to *SFTPA2* mutations and characterized by pulmonary fibrosis, interstitial pneumonia and lung cancer.

Finally, pulmonary fibrosis can also be part of the POIKTMP syndrome (poikiloderma with tendon contractures, myopathy and pulmonary fibrosis). FPF can manifest either as IPF, but also as connective tissue disease-interstitial lung disease (CTD-ILD) or unclassifiable interstitial lung disease (U-ILD) [7,11].

On the other hand, there is little evidence of the possible role of genetics in the etiology of sporadic IPF, although mutations in FPF-causing genes are also found in about 10–20% of sporadic IPF cases.

Even if the genetic predisposition to PF is known, the molecular diagnosis of familial cases is probably underestimated, and this could be related to the incomplete penetrance of the disease or the lack of information from the patient’s pedigree. In order to recognize the familial cases, it is important to identify the risk in family members not only for PF but also for possible related conditions, mainly lung cancer and bone marrow failures. In addition to the Mendelian forms, several studies support the existence of a genetic predisposition to pulmonary fibrosis, which is expressed as a complex trait in which low-penetrance genes and environmental factors play a role. In particular, the involvement, even in sporadic forms, of genes related to the production of surfactant proteins, which would alter the functionality of the pulmonary alveoli, or of genes responsible for an increased chronic secretion of mucus, is highlighted [12].

Finally, epigenetic alterations seem to play a role in fibrogenesis. Several studies consider cellular aging as an endogenous causative factor of IPF, which favors the development of genomic instability, the establishment of epigenetic alterations, the shortening of telomeres and cellular senescence, thus laying the foundations for the development of fibrosis [13].

Overall, heterogeneous genetic variants could contribute to the development of an altered broncho-pulmonary tissue, which would become more susceptible to recurrent micro-lesions from various potential environmental factors.

In this study, a state-of-the-art review of the genetic and epigenetic bases already explored in IPF and FPF will be presented, along with an overview of their possible implications for therapy. For this purpose, a non-systematic, narrative literature review was conducted. The search engine PubMed was adopted to retrieve the most relevant articles on these topics from international scientific journals.

## 2. The Genetic Basis of Idiopathic Pulmonary Fibrosis and Familial Pulmonary Fibrosis

**Figure 1:**
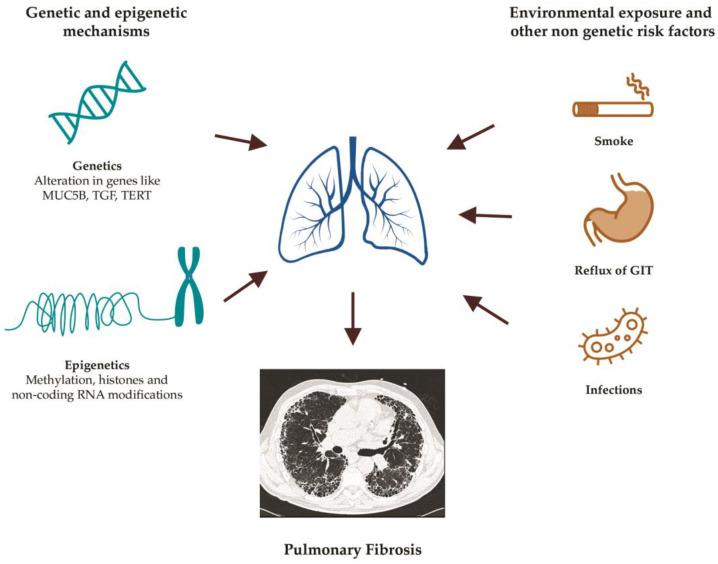
Main risk factors for the development of pulmonary fibrosis.

PF can have a strong genetic component in 10–20% of cases, as previously described, or gene variants can modify the risk so that it is affected together with the influence of the environment. In addition, genetic factors might also influence the onset of the disease [14] (Figure 1).

Particularly, mutations in the genes of the telomerases, such as *TERT*, *TERC*, *PARN* and *RTEL1*, which regulate the telomere length, thus resulting in shortened telomeres, have a recognized causative role in IPF and FPF [15].

A growing number of genetic factors predisposing to pulmonary fibrosis have been explored, also thanks to the possibility of conducting genome-wide association studies (GWAS). Particularly, sequence variants in genes for the surfactant proteins causing surfactant dysfunction (*SFTPC*, *SFTPA1*, *SFTPA2*, *ABCA3*), or polymorphisms in *MUC5B*, causing overexpression of the mucin 5B, or in *TOLLIP* (an inhibitor of the Toll-like receptors 2 and 4) together to mitochondrial dysfunction are other genetic mechanisms associated with an increased risk of IPF [16].

Finally, epigenetic mechanisms can modify the expression of genes implicated in the development of IPF through DNA methylation, histones and non-coding RNA modifications [17], as well as a result of exposome triggers.

**Figure 2:**
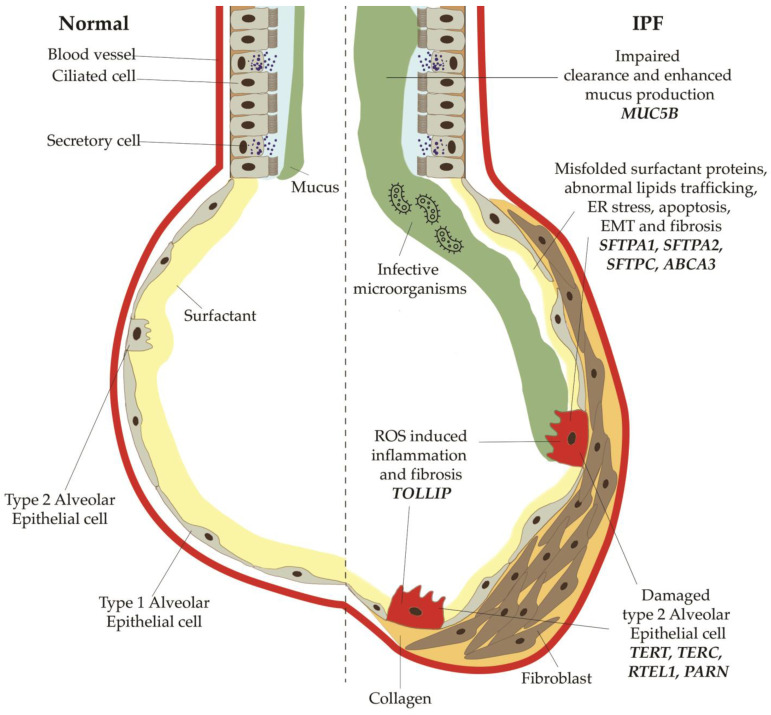
Main profibrotic mechanisms secondary to mutations or polymorphisms in the genes for telomerases, surfactant proteins, mucin 5B. Mutations in *TERT*, *TERC*, *PARN* and *RTEL1* decrease the activity of telomerase, thus resulting in augmented shortening of telomeres. *SFTPC*, *SFTPA1*, *SFTPA2*, *ABCA3* normally modulate and stabilize the alveolar surfactant tension; when altered, they can induce an increased stress of the endoplasmic reticulum, which finally lead to epithelial-mesenchymal transitions and apoptosis of type 2 alveolar cells. Polymorphisms of *MUC5B*, a regulatory mucin, are responsible of mucociliary disfunction, with impaired clearance and enhanced mucus production, which predisposes to bacterial overgrowth and infections.

Table 2 summarizes the principal genes described in IPF and FPF and their profibrotic mechanisms, which are also depicted in Figure 2. While mutations in *TERT*, *TERC*, *PARN* and *RTEL1* decrease the activity of telomerase, thus resulting in augmented shortening of telomeres, variants of surfactant proteins (*SFTPC*, *SFTPA1*, *SFTPA2*, *ABCA3*), which normally modulate and stabilize the alveolar surfactant tension, can induce an increased stress in the endoplasmic reticulum, which finally leads to epithelial-mesenchymal transitions and apoptosis of type 2 alveolar cells.

All the reported causative genes for PF show an autosomal dominant pattern of transmission, leading to a 50% chance of transmitting the pathogenetic variant to the offspring. However, the expression of the phenotype is influenced not only by incomplete penetrance, intrafamilial clinical variability and environmental factors but also by the pathomechanism related to the specific gene involved. For example, for genes involved in telomere maintenance, it is reported that the phenomenon of anticipation, is possibly favored by epigenetic mechanisms triggering telomere shortening [18].

Moreover, polymorphisms of *MUC5B*, a regulatory mucin, are responsible for mucociliary disfunction with impaired clearance and enhanced mucus production, which predisposes to bacterial overgrowth and infections.

### 2.1. Telomere Dysfunction

Telomere dysfunction is one of the main drivers of IPF, as many sequence variants in telomere genes have been described in IPF [19,20,21,22,23,24,25,26,27,28,29,30,31,32,33,34,35,36,37,38,39]. In eukaryotic cells, telomeres are ribonucleoprotein structural complexes that are located at chromosomal ends. Their role is to protect the integrity of the genome by preventing chromosome degradation [40]. Telomere structure is composed of tandem DNA repeats and is protected by shelterin complexes, which, in cases of deficiency or ablation, can contribute to telomere dysfunction [41]. It is known that at every cell cycle and division, telomeres naturally shorten. To counteract this shortening process, some cells express telomerase, whose main components are the catalytic subunit telomerase reverse transcriptase (*TERT*) and telomerase RNA component (*TERC*). Other accessory components act to stabilize TERC, such as dyskerin, NHP2, NOP10 and GAR1 [42]. The length of telomeres is fundamental in regulating cellular senescence or apoptosis [43,44].

Among the Mendelian telomere disorders, PF is the most common in the adult age, indeed numerous mutations in telomere-related genes have been described in association with IPF [21,22,23,24,25,26,27,28,29,30,31,32,33,34,35,36,37,38,39,40,41]. Although the exact mechanisms that lead to fibrogenesis in patients with mutations in telomere-related genes are not fully described, it seems that a key role might be played by alveolar stem cells’ cellular senescence and death induced by telomere dysfunction [45].

Whilst 15% of FPF present mutations in *TERT* and *TERC* genes, these mutations can be found also in 2% of sporadic IPF. *TERT* and *TERC* mutations are mainly heterozygous (related to an inherited or *de novo* mutation), thus showing a dominant transmission. It has been hypothesized [21,27] that as many as 25% of sporadic IPF and 37% of FPF might have shorter telomeres. Indeed, other telomere mutations, such as *PARN* or *RTEL1*, should be considered as risk factors for the development of earlier IPF onset and more rapid progression and thus the risk of death.

Since the decreased ability to regenerate damaged or injured lung tissue was experimentally demonstrated in mice models of IPF with TERC mutations (*TERC* KO mice), it was hypothesized that adult stem cells of the lung, the alveolar epithelial type 2 cells (AEC type 2), are the most affected by shortening of the telomeres [46]. In this mice model, AEC type 2 are significantly reduced, as they are more vulnerable to apoptosis and cellular senescence [47], thus contributing to fibrogenesis. This process has been partly due to the lack of generation of new alveoli and partly secondary to the consequent increase in mechanical stress and tension [48]. More in detail, AEC type 2 senescent cells release proinflammatory and pro-fibrotic cytokines and growth factors, such as transforming growth factor beta (TGF-β), interleukin-1 (IL-1), interleukin-6 (IL-6) [49], which induce the infiltration of innate immune cells, neutrophils and macrophages that are experimentally detected in BAL of mice models [50]. Infiltrated macrophages too contribute to fibrogenesis through the secretion of profibrotic interleukin-10 (IL-10) and TGF-β [51].

In summary, apoptotic or senescent lung AEC type 2, in which telomere mutations have occurred, drives pulmonary fibrosis firstly via the lack of ability to regenerate healed tissue and to generate new alveoli, thus inducing an increase in mechanical tension and fibrosis, and secondly via the cytokine-induced recruitment of inflammatory cells, fibroblast and myofibroblasts. Although AEC type 2 cells are the most affected cells, telomere dysfunction might involve other stem cells such as pulmonary club cells and basal cells.

### 2.2. MUC5B

*MUC5B* encodes a precursor protein of the mucin 5B, a major gel-forming mucin that is secreted by proximal submucosal glands and distal airway secretory cells. This protein is involved in lung mucus production, homeostasis and the immune regulation of bronchoalveolar epithelial function. Genetic variants of *MUC5B*, causing its overexpression in the bronchoalveolar epithelium, are recognized as one of the main risk factors for IPF, although the specific mechanism, which is involved in the induction of IPF is not already clear [52]. Particularly, the presence of a single nucleotide polymorphism (SNP) for the minor allele T of *MUC5B*, which is located at 3 kb upstream of the *MUC5B* gene transcription start site on 11p15 (rs35705950), has been described as the strongest risk factor to predispose to IPF both in sporadic and familial cases [53,54]. This gain of function mutation causes the overexpression of *MUC5B,* which is indeed associated with the failure of the reparative and regenerative mechanisms of the alveoli, mucociliary dysfunction and pollutants retention, which are the basis for the development of honeycombing cysts and fibroproliferative degeneration [55,56]. Excessive release of mucin 5B has been also associated with the exacerbation of IPF. Moreover, the risk of developing IPF increases in homozygous carriers for the T allele rs35705950 (TT), with an odds ratio for IPF of 21.8 versus 9 for heterozygous [53].

The frequency of the T allele in the general population and in cohorts of IPF patients has been thoroughly investigated in several studies across the world. A genome-wide association study (GWAS) has found that in the Caucasian population 41.9% of IPF patients and 10.8% of the healthy control group are carriers of the T allele of the rs35705950 [57]. On the contrary, the frequency of the T allele is far lower in Asiatic and mixed populations, at about 3.33% in IPF patients and 0.66% in healthy people [58]. A recent meta-analysis based on 24 case-control studies including 6749 IPF patients and 13.898 healthy controls confirmed that the presence of the T allele confers susceptibility to develop IPF and that the risk is greater in people carrying two copies of the T allele (OR for TT genotype 10.12 vs. 4.84 for GT genotype) [59]. Quite unexpectedly, the GG wild-type genotype seems, however, linked to a worse overall survival than TT or GT genotypes [60]. These results have been confirmed also in a recent study by Biondini et al. [61], which specifically focused on IPF patients in antifibrotic therapy (GG 42 versus TT/GT 74 months overall survival). It has also been reported that rs35705950 T is associated with the prevention of bacterial burden in the bronchoalveolar lavage of IPF patients and a lower decline in respiratory function [62]. Further studies are needed to explain why rs35705950 T confers a survival advantage in IPF despite increasing the risk of developing the disease. Probably, the interaction of this common variant with other environmental and epigenetic factors might be the reason, which has to be further investigated.

### 2.3. TOLLIP

The Toll-interacting protein TOLLIP acts as an inhibitor of the Toll-like receptors (TLRs) 2 and 4, which are active in the lung. As a consequence, TOLLIP contributes to the suppression of tumor necrosis factor-α (TNF-α) and IL-6 production [63]. Several studies have reported that genetic variations of TOLLIP can influence the development and prognosis of IPF and FPF [9]. Particularly, the minor allele rs3750920 seems to influence the response to IPF treatment through a decrease in the expression of TOLLIP mRNA, while the minor allele rs5743890 seems to be associated with IPF progression through an impaired production of TOLLIP [64].

TOLLIP is mainly expressed in the lung by alveolar type II cells, macrophages and basal cells, where it can prevent damage from reactive oxygen species (ROS) induced by mitochondrial damage. Moreover, it can protect bronchial cells and modulate inflammation and autoimmunity via different pathways (TLR, TNF- α, IL-1β, IFN- β, IL-13) [65,66].

Consistently, IPF outcome might be influenced by SNPs in the TOLLIP gene. Bonella et al. recently found that IPF patients carrying the TOLLIP rs5743890 minor allele had a worse prognosis, with a median reduction in survival of 20 months compared to IPF patients without this mutation [67]. Moreover, not only homozygous but also heterozygous patients showed a more rapid progression of the disease, with a greater decline in FVC over time. The authors thus proposed that the minor allele TOLLIP rs5743890 a possible role as a genetic biomarker of progression to be evaluated in the risk stratification of IPF. The TOLLIP rs3750920 polymorphism instead seems to influence the response of IPF to treatment, particularly a study by Oldham et al. in the pre-antifibrotic era that showed better results with N-Acetylcysteine treatment in carriers of this SNP [64].

### 2.4. SFTP and ABCA3

AEC type 2 are responsible for the production of the surfactant. Particularly, the surfactant is composed of phospholipids and the following four surfactant proteins: SP-A, SP-B, SP-C and SP-D. Surfactant proteins are synthesized in the endoplasmic reticulum (ER) of AEC type 2, from which they are transported and subsequently stored in the lamellar bodies. Finally, these proteins are secreted in the alveolar space [68,69].

In the literature, it has been described that several mutations of the genes encoding these proteins (*SFTPA1*, *SFTPA2*, *SFTPC* and *ABCA3*—ATP binding cassette subfamily A member 3) are associated with FPF [70,71,72,73,74]. The mutations affecting the genes for the surfactant proteins, although very rare, are highly penetrant and have a great effect on affected people, causing severe forms of pulmonary fibrosis [55].

In detail, variants in the *SFTPA2* gene (the surfactant protein A) are associated with ER stress and an increased risk of developing lung cancer in smokers [71]; those in the *SFTPC* gene (the surfactant protein C) lead to a misfolded SP-C, which thus accumulates in the ER, causing ER stress. Particularly, the BRICHOS domain, critical for the SP-C proper folding, is usually the most affected by *SFTPC* mutations [68,75].

Finally, rare variants in the gene encoding for the protein ABCA3 have been described as a risk factor for pulmonary fibrosis. ABCA3 is a protein, which in AEC type 2 acts as a transporter protein for lipids through the plasma membranes [76,77]. When mutated, the protein shows abnormal trafficking and functionality, which causes ER stress due to a higher lipids retention in the ER. These processes activate apoptosis in AEC type 2 and fibrogenesis [78]. Whilst mutations of *SFTPA2* and *SFTPC* are dominant, *ABCA3* mutations are causative of a recessive form of the disease [79].

The possible mechanism at the basis of fibrogenesis in patients carrying mutations in the genes encoding for surfactant proteins might rely on the activation of epithelial-to-mesenchymal transition (EMT) induced by ER stress. Indeed, ER stress is able to activate epithelial cell differentiation in mesenchymal cells and AEC type 2 apoptosis. This causes the production and deposition of collagen and the subsequent fibrosis in an alveolar environment, which is poor for functional surfactant proteins [80,81,82].

## 3. The Epigenetic Influence in Idiopathic Pulmonary Fibrosis and Familial Pulmonary Fibrosis

Besides genetics and genomics, which help characterize the molecular landscape of IPF and FPF, a major role is also played by epigenetics. Several studies showed indeed that multiple genes are differentially expressed in IPF lung, mainly involved in pathways such as TGF-β signaling, epithelial to mesenchymal transitions, fibroblast proliferation [83,84,85]. Gene expression is controlled by several epigenetic mechanisms, which coordinate the activation or silencing of gene transcription. Epigenetic modifications can be essentially grouped into the following three types: DNA methylation of CpG sites, histone post-translational modifications and noncoding RNAs. Epigenetics impacts gene expression modulation independently of the DNA sequence. Developmental processes and environmental factors, such as behavioral habits, diet and drugs broadly defined as exposome, and aging can trigger epigenetic modifications, thus influencing gene expression.

All the biological features characterizing PF could be explained by gene expression dysregulation related to epigenetic modifications. Considering the dynamic nature of epigenetic modifications, they represent an attractive druggable target, since epigenetic marks can be reverted by specific treatments, such as histone deacetylases inhibitors (HDACi) [86].

Moreover, distinct episignatures are, indeed, emerging to be disease-specific and the epigenetic profile may be exploited to support the clinical diagnosis. The identification of methylation changes associated with disease development is particularly important for pathologies strongly associated with environmental exposure, as in the case of PF. Epidemiological studies have, indeed, demonstrated the association between exposure to inhaled environmental agents and IPF development, including cigarette smoke, wood dust, metal dust, silica, textile dust and possibly agriculture, farming and livestock [87,88]. Cigarette smoke, in particular, is considered the strongest risk factor for the development of disease, also for FPF, thus suggesting that, also in cases with a genetic predisposition to the disease, cigarette smoking contributes significantly to its development, possibly acting at the epigenetic level [89]. Genome-wide methylation studies on IPF are ongoing in order to identify specific altered methylation patterns that may shed light on the effect of environmental exposures and on the pathomechanisms underlying PF development. Epigenetic signatures may be potential biomarkers to support the clinical diagnosis, identify novel pharmacological therapies to revert epigenetic changes and monitor the effect of already available therapies.

### 3.1. DNA Methylation

Human DNA is enriched in CG dinucleotides, especially in the regulatory regions such as gene promoters. These CpG sites can undergo the addition of a methyl group to the cytosine residue. This methylation process is regulated by specific enzymes, essentially DNA methyltransferases (DNMTs), and the presence of methylated CpGs blocks the binding of the RNA polymerase complex to the promoter region and thus silences gene expression. DNA methylation is an epigenetic mark that triggers conformational chromatin changes leading to different expression levels of genes; the general rule is that hypomethylation in gene promoters should result in increased expression of the related genes, while hypermethylation causes a decrease in expression levels. This rule is not always valid if methylation is located in other regulatory genome regions, such as enhancers [90].

Several genome-wide methylation studies reported multiple differentially methylated regions (DMRs) between IPF lung and healthy controls [91]. The majority of these DMRs are located outside gene promoters or other known regulatory elements, making it tricky to assign a biological meaning; however, some studies in IPF lungs reported different DNA methylation levels in the promoters and enhancers of genes known to be involved in the regulation of important cellular processes impaired in IPF, such as *MUC5B*, *PTGER2* (Prostaglandin E receptor 2) and *THY1* (Thy-1 antigen).

Regarding *MUC5B* and the rs35705950 risk allele, Helling and colleagues [92] identified a highly conserved *MUC5B* enhancer region encompassing the rs35705950 variant. This enhancer element dynamically binds the transcription factor FOXA2 as well as RNA polymerase II, suggesting its important role in controlling MUC5B expression. Indeed, the authors indicated that increased methylation is associated with increased expression of MUC5B, IPF and the risk allele T at rs35705950.

Prostaglandin E_2_ (PGE_2_) pathways are essential to maintain the correct homeostasis of fibroblasts, guaranteeing the appropriate apoptosis. The lungs of IPF patients are substantially resistant to PGE_2_, which is conditioned by reduced expression of its receptor, E prostanoid 2 (EP_2_), due to DNA hypermethylation of its promoter, *PTGER2* [93]. The authors showed that treatment with the DNA methylation inhibitors 5-aza-2’-deoxycytidine or zebularine as well as DNA methyltransferase-specific siRNA can restore EP_2_ mRNA transcription and thus the PGE_2_ responsiveness in fibrotic fibroblasts but not in nonfibrotic controls.

Fibroblastic foci typical of IPF are characterized by abnormal myofibroblasts, in which gene expression is completely altered, especially for several “IPF suppressor genes” [94,95,96,97]. Among these, *THY1* encodes for the thymocyte differentiation antigen 1 (Thy-1), a cell outer membrane glycoprotein also known as CD90. Studies in human and murine lung specimens demonstrated that the absence of Thy-1 promotes myofibroblast differentiation and that this is due to hypermethylation of the *THY1* promoter [94,98]. The increased methylation of *THY1* seems to be related to hypoxia [99], and therapy with 5-aza-2’-deoxycytidine is able to restore Thy-1 expression in primary mouse lung fibroblasts and human lung fibroblasts [100,101].

These studies suggest that alteration of DNA methylation at genomic loci closely related to the pathogenesis of IPF is a crucial factor in the development of IPF and that DNMT-targeted therapies could be a resource for remodeling this process, although further studies on their specificity and adverse effects are needed. Little is known about the role of epigenetic modifications in the development of FPF; nevertheless, environmental exposure can induce disease-specific methylation changes associated with increased risk of disease development, also in familial cases, thus suggesting a role for epigenetic changes, in addition to the genetic predisposition, in FPF [89].

### 3.2. Histone Modification

In addition to DNA methylation, remodeling of the chromatin state is also regulated by post-translational modifications of histones that change the conformation of the chromatin complex, leading to different levels of condensation and thus affecting the gene expression of related loci. Histones can be modified through different mechanisms, among which the two most common are methylation and acetylation. Both types of modifications are dynamically regulated by different classes of enzymes responsible for the addition or removal of methyl or acetyl groups from specific histone residues. Histone methylation is controlled by the following two sets of enzymes: histone methyltransferases (HMTs) and histone demethylases (HDMs), while histone acetylation is the product of the dynamic interplay between histone acetyltransferase (HATs) and histone deacetylase (HDAC).

Among the enzyme alterations responsible for histone modifications in the pathogenesis of IPF are increased activity and expression of EP300. EP300 encodes for a histone acetyltransferase involved in cell proliferation and differentiation. Rubio and colleagues [102] demonstrated that in the nuclei of IPF patients, EP300 hyperactivity inhibits nuclear HDAC1 and impairs the functionality of MiCEE, a ribonucleoprotein complex that mediates global genome organization. Inactivation of EP300 in vitro (patient-derived primary fibroblast), in vivo (bleomycin mouse model) and ex vivo (precision-cut lung slices, PCLS) IPF models causes reduction of fibrotic hallmarks, making EP300 inhibition a novel potential therapy against IPF.

Aberrant HDAC activities are also involved in the epigenetic silencing of Thy-1, an already mentioned “fibrotic suppressor surface glycoprotein”. Indeed Thy-1 silencing is regulated not only by promoter hypermethylation but also by histone modifications in lung fibroblasts [103] and treatment with the histone deacetylase class I and class II inhibitor trichostatin A (TSA) is able to completely reduce Thy-1 expression, better than treatment with 5-aza-2’-deoxycytidine to reduce DNA promoter hypermethylation. Moreover, TSA treatment is also able to prevent the smooth muscle α-actin (α-SMA) transcription and translation. This cytoskeletal protein is usually expressed in myofibroblasts derived from TGFβ1-mediated differentiation of fibroblasts, a key process of IPF pathogenesis [104].

These studies highlight the close interaction between DNA methylation and histone modification and how treatments against the enzymes responsible for regulating these epigenetic mechanisms could be useful in the treatment of IPF.

Other emerging pathways involved in the development of the lung, and which seem to be epigenetically influenced in IPF patients are the Hedgehog pathway GLI1 and the WNT pathway of beta-catenin [105].

### 3.3. Non-Coding RNA Gene Silencing

Genes expression can also be controlled by microRNAs (miRNA), non-coding RNAs composed of 17–25 nucleotides. They interact with the 3′ untranslated region of mRNAs mediating mRNA degradation and gene silencing.

Several miRNAs expression profile studies showed that alteration in the expression of specific miRNAs affects fibroproliferation, epithelial-mesenchymal transition and the TGF-β1 signaling pathway [106,107,108,109]. MicroRNAs are differently upregulated or downregulated during the phases of IPF pathogenesis, and, consistently, both antifibrotic and profibrotic miRNAs were identified. They can modulate the fibrogenesis in IPF patients, altering biological processes such as collagen production and deposition, differentiation of fibroblast to myofibroblast and EMT [17].

Intriguingly, miR-21 plays a key role in IPF. Indeed, miR-21 can stimulate TGF-β thus upregulating the EMT and impairing earlier the forced vital capacity [110]. Moreover, miR-34 is overexpressed in IPF, regulating the response of epithelial cells and several modulators of miRNA expression are the subject of research, since they could influence the prognosis and the pathogenesis in IPF [110].

Emerging studies are focusing on the role of long non-coding RNAs (lnc-RNAs) in fibrogenesis, such as an antifibrotic role for FENDRR [111] and a profibrotic role for DNM3OS [112]; however, their role in the pathogenesis of pulmonary fibrosis is still debated.

As for methylation changes, studies on ncRNAs have been mainly focused on IPF. However, the role of these RNAs may be played also in FPF, as an additional mechanism influencing the onset and the severity of the condition associated with exposome [113].

## 4. Idiopathic Pulmonary Fibrosis and Lung Cancer: A Common Genetic Background?

Some genes associated with the onset and progression of IPF might support clonal selection and favor cancer transformation.

Interestingly, genes supporting the inflammation and fibrogenesis in IPF, such as *TGF-β1*, interleukin-1 receptor alpha (*IL1RN*), interleukin 8 (*IL-8*), Toll-like receptor 3 (*TLR3*), telomerases, tumor protein 53 (*TP53*), are also associated with cancer. While *TP53* can act as an oncogenic driver, the others can sustain neoplastic transformation but cannot induce it. The *TP53* gene was found to be significantly mutated in cases of lung cancer associated with IPF. Mutations in the JAK-STAT signaling pathway were also significantly amplified in IPF-associated lung cancer [114].

As already described, mutations in the telomerase genes are one of the main genetic risk factors for pulmonary fibrosis, but the angiogenic and pro-invasive properties they confer could be adopted by yet-transformed malignant cells [115]. Moreover, numerous risk factors (i.e., cigarette smoke and air pollution) are shared between IPF and cancer.

Intriguingly, the thyroid transcription factor-1 (TTF-1), a highly specific biomarker of primary adenocarcinoma of the lung, seems to be implicated also in the pathogenesis of IPF. *TTF-1* is mainly expressed in APC type 2, and it regulates the expression of surfactant proteins in the lungs, which are fundamental for the homeostasis of the alveolar *milieu* [116]. *TTF-1* is an oncogene, which might contribute to cell proliferation in cancer and fibrosis since its expression is enhanced in injured lungs [117].

A link between IPF and lung cancer secondary to mutations in the genes of the surfactant proteins has begun to be investigated. Polymorphisms of *SFTPA1* and *SFTPA2*, indeed, predispose to IPF through ER stress and consequent alterations in APC type 2, whilst pathogenetic variants of *SFTPA2* are responsible for ILD2, an autosomal dominant condition characterized by a strong genetic predisposition to lung cancer. By promoting cell proliferation and fibrogenesis, these mutations can also induce neoplastic transformation [71]. Similar mechanisms have been described for mutations of *SFTPC* [118]. *ABCA3*, which is well known for its profibrotic action when mutated, is also implicated in the onset and progression of lung cancer and acts as a mediator of chemoresistance in lung neoplasm [119].

Even if the mechanisms of their role in lung cancer and pulmonary fibrosis are still not well defined, the peroxisome proliferator-activated receptors (PPARs) nuclear receptors seem to play a part, since their deregulation has been described in several inflammatory conditions, such as cancer and atherosclerosis, but also in IPF. PPARs are involved in the regulation of the metabolism of lipids and in cellular proliferation [120].

Another link between IPF and lung cancer resides in the matrix metalloproteinase 1 (*MMP1*), which shows a high rate of genetic mutations in both of these two pathologies. MMP1 is well represented in neoplasms, promoting the spreading of metastases and their invasive potential, and enhancing the EMT in pulmonary fibrosis, angiogenesis and cell migration. Consistently, high levels of MMP1 have been found in the early phases of lung cancer developed in the context of pulmonary fibrosis [121].

Moreover, genome-wide methylation studies as well as gene expression studies showed several parallelisms between the pathogenetic mechanisms that drive IPF and lung cancers. Rabinovich et al. reported that about 402 differentially methylated CpG islands in IPF lung overlap those altered in lung cancer [122]. A recent study [123] showed that the gene expression of twenty genes in stromal cells from patients with IPF and lung adenocarcinoma (ADC) was similarly up- or down-regulated compared to control samples. This suggested a slight overlap between the etiopathogenetic processes that drive IPF and lung cancer.

In conclusion, several mechanisms seem to be shared between pulmonary fibrosis (both idiopathic and familial) and lung cancer, ranging from mutations and polymorphisms of typical genes involved in the fibrogenesis to alterations in oncogenes with a novel potential profibrotic action. Intriguingly, nintedanib, a tyrosine kinase inhibitor currently approved for the treatment of idiopathic pulmonary fibrosis, has been inherited from oncology, underlying again the common background of pulmonary fibrosis and cancer.

## 5. The Interplay between Genetics and Antifibrotic Therapy

A recent study based on next-generation sequencing (NGS) and bioinformatics described some genetic and epigenetic pathways that can be influenced by nintedanib, an antifibrotic therapy [124].

Nintedanib is a tyrosine kinase inhibitor, which exerts its antifibrotic effects by targeting and interfering with fibroblast-derived growth factor receptor (FGFR), platelet-derived growth factor receptor (PDGFR), vascular endothelial growth factor receptor (VEGFR) and potentially inhibiting the transforming growth factor β (TGF-β) signals to downregulating the ECM [3]. The authors explored with NGS the mRNA and miRNA changes in primary human IPF fibroblasts treated with 2 μM and 4 μM nintedanib, compared to fibroblasts not exposed to treatment. They found 157 downregulated and 151 upregulated genes in the nintedanib-treated fibroblasts. Most of them were active in the complex biological pathways of the cell cycle, cell division, DNA replication and mitotic nuclear division.

Notably, after nintedanib treatment, the authors identified four downregulated and one upregulated genes, which act on the following miRNA-mRNA interactions: *E2F1*, *NPTX1*, *DDX11*, *PLXNA4* (downregulated) and *SLC25A23* (upregulated).

E2F1 is a critical transcription factor in the cell cycle and apoptosis, particularly because it is required for the S phase of the cell cycle. By increasing the levels of E2F1, the proliferating and profibrotic effects of VEGF, epidermal growth factor (EGF) and keratinocyte growth factor (KGF) can be exerted. Nintedanib manages to reduce the levels of E2F1 in the treated fibroblasts, and this might lead to slow fibrogenesis in IPF.

NPTX1 belongs to the family of neuronal pentraxin [125], thus its downregulation should not have a significant effect in the lung, where, on the contrary, pentraxin-2 seems to play a beneficial role in preventing fibrosis and a global clinical trial is ongoing to validate a new antifibrotic therapy based on recombinant human pentraxin-2 (rhPTX-2) [126].

DDX11 promotes cell proliferation and metastasis in tumoral cells, whilst it might induce the proliferation of fibroblasts in IPF. Nintedanib manages to suppress the DDX11 signaling, thus inhibiting fibroblast’s proliferation.

PLXNA4 is also downregulated by nintedanib treatment. Since its action is to promote the vascularization of the tumors, induced by VEGF, its downregulation can decrease the angiogenesis in IPF.

Finally, SLC25A23, which, in contrast, is upregulated by nintedanib, induces cell death through oxidative stress. It has been hypothesized that upregulating SLC25A23, nintedanib can reduce fibroblast proliferation, induce their apoptosis and decrease angiogenesis in IPF.

Recently, another interesting study focused on the efficacy and safety of nintedanib and pirfenidone in a group of patients with pulmonary fibrosis carrying mutations in telomerases genes. In this work, authors found that both antifibrotic treatments were associated with a reduced decline of FVC, without observing unexpected adverse events [127].

Moreover, another study demonstrated that also pirfenidone can slow the rate of progression of EMT modulating several gene-induced pro-fibrotic pathways [128]. Pirfenidone can suppress enzymes involved in EMT, such as SULF2, and upregulate antifibrotic genes, such as Gremlin 2 (GREM2), which subsequently induce the repair of the injured alveolar epithelium through fibroblast growth factor 10, thus preventing fibrosis. Moreover, EDN1 and 5-HTR2B, two genes with profibrotic action linked to collagen deposition and fibroblast proliferation, are also downregulated by pirfenidone.

Since available drugs are not able to cure IPF, several studies have addressed the use of gene therapies as potential approaches to attenuate a wide range of processes involved in fibrosis. To date, despite the potential benefits of gene therapy, no clinical trials for treating IPF have not been launched. Developing novel drugs to treat IPF is, indeed, challenging due to the complex pathogenesis of the disease and the difficulty of modeling the disease in animals. Currently, available animal models are not specific for IPF but only recapitulate some aspects of pulmonary fibrosis artificially induced by different chemicals (e.g., bleomycin, FITC and lipopolysaccharide). Initial studies exploring the potential application of gene therapies to IPF focused on inducing targeted gene overexpression using both nanoparticles and viral vectors [129,130]. This approach mainly targeted inflammatory pathways, including the TGF-beta and FGF7 signaling [131,132].

More recently, the use of gene silencing by siRNA (small interfering RNA) for treating PF was investigated in several studies that described the efficacy of multiple siRNAs combined with antifibrotic compounds in treating several aspects of PF [133]. Very few studies have examined the use of miRNAs to induce gene expression silencing in PF [134,135]. These studies suggested that a miRNA-based therapy may have great potential in repressing multiple fibrosis-related genes simultaneously. However, the pleiotropic effect of miRNAs on different gene transcripts (not all characterized yet) raises safety concerns on the therapeutic use of these ncRNA.

Altogether, these studies support the applicability of gene therapy to suppress the progression of fibrosis. However, no gene therapy approach has demonstrated the capability to reverse established fibrosis.

## 6. Outlook and Perspectives

Nowadays, the possibility of exploring the genetic and epigenetic basis of pulmonary fibrosis more in depth represents a clinical and scientific challenge that could, in the near future, help in both the diagnosis and the development of targeted therapy for this devastating disease.

Hopefully, genetics might soon support the diagnostic algorithm of IPF, integrating data from radiology and pathology at the molecular level. To this end, it is essential to underline the role the genetic counseling in referring to genetic analysis of the population at risk of familial forms (i.e., asymptomatic relatives of patients carrying known mutations of FPF), and in the correct interpretation of the considerable amount of data that can be made available by genome-wide array genetic analysis.

Reversing the epigenetic changes capable of influencing the genesis and progression of pulmonary fibrosis might be an interesting research perspective, as well as designing trials for targeted therapy at the genetic level.

Since IPF and PFF are rare diseases, it is essential that knowledge of the genetic basis of pulmonary fibrosis should be shared with all other healthcare personnel involved in the management of ILD.

## 7. Conclusions

An increasing role of genetics and epigenetics in the pathogenesis and prognosis of IPF and PFF is emerging. As described above, specific Mendelian mutations in telomere genes are able to determine the development of familial forms of pulmonary fibrosis, with an earlier onset and often a more severe prognosis. Likewise, gene variations capable of altering surfactant proteins are known to be responsible for the fibrosing disease, some of which affect children. Finally, polymorphisms of mucins, i.e., MUC5B or Toll-like receptor inhibitors (TOLLIP), have been associated with a greater risk of developing pulmonary fibrosis.

Many of the genes and pathways involved in pulmonary fibrosis development are today known because of their involvement in the Mendelian form and GWAS studies. Beyond the Mendelian forms, many of the same loci and pathways may be affected by variants or epigenetic modifications that can modify the risk of diseases in the presence of environmental triggers.

Although the genetic predisposition to pulmonary fibrosis is known, the molecular diagnosis of familial cases is probably still underestimated, and this could be related to the incomplete penetrance of the disease or the lack of information from the patient’s pedigree. In order to recognize the familial cases, it is important to identify the risk in family members not only for pulmonary fibrosis but also for possible related conditions, such as lung cancer and bone marrow failures. 

In addition, it is emerging that epigenetic mechanisms can affect the development and prognosis of pulmonary fibrosis. DNA methylation of CpG sites, histone post-translational modifications and non-coding RNA gene silencing are mechanisms that should also be explored in fibrogenesis.

Interestingly, epigenetic signatures of disease can have a clinical utility as biomarkers or druggable targets since epigenetic marks can be reverted.

Of interest, genes supporting the inflammation and fibrogenesis in IPF, such as TGF-β1, interleukin-1 receptor alpha (IL1RN), interleukin 8 (IL-8), Toll-like receptor 3 (TLR3), telomerases, tumor protein 53 (TP53), are also associated with cancer and genome-wide methylation studies, as well as gene expression studies, showed several parallelisms between the pathogenetic mechanisms that drive IPF and lung cancers. Further studies are needed to better elucidate the molecular mechanisms of the relationship between pulmonary fibrosis and lung cancer.

Finally, data are emerging on the molecular pathways, at both the genetic and epigenetic levels, which form the basis of the efficacy of the antifibrotic therapies.

## Figures and Tables

**Table 1:** Causative genes for FPF. The relative proteins, OMIM identifier, phenotype and type of inheritance are described.

Gene	Protein	Disease (OMIM)	Phenotype	Inheritance
*TERT*	Telomerase reverse transcriptase	PFBMFT1 (614742)	Pulmonary fibrosis and/or bone marrow failure, telomere-related	AD
*TERC*	Telomerase RNA component	PFBMFT2 (614743)	Pulmonary fibrosis and/or bone marrow failure, telomere-related	AD
*RTEL1*	Regulator Of Telomere Elongation Helicase 1	PFBMFT3 (616373)	Pulmonary fibrosis, adult onset	AD
*PARN*	Poly(A)-Specific Ribonuclease	PFBMFT4 (616371)	Pulmonary fibrosis, adult onset	AD
*ZCCHC8*	Zinc Finger CCHC-Type Containing 8	PFBMFT5 (618674)	Pulmonary fibrosis and/or bone marrow failure, adult onset	AD
*RPA1*	Replication Protein A1	PFBMFT6 (619767)	Pulmonary fibrosis and/or bone marrow failure and skin abnormalities	AD
*SFTPA2*	Surfactant Protein A2	ILD2 (178500)	Pulmonary fibrosis, interstitial pneumonia, lung cancer	AD
*FAM111B*	FAM111 Trypsin Like Peptidase B	POIKTMP (615704)	Poikiloderma, hereditary fibrosing, with tendon contractures, myopathy, and pulmonary fibrosis	AD

**Table 2:** Genes described in idiopathic pulmonary fibrosis (IPF) and familial pulmonary fibrosis (FPF) and their profibrotic mechanisms.

Gene ^1^	Main Function	Profibrotic Mechanism
*TERT*	Telomerase	Decreased activity of telomerase
*TERC*	Reverse Transcription in Telomerase	Decreased activity of telomerase
*PARN*	Stability of mRNA in Telomerase	Shortening of telomeres
*RTEL1*	DNA helicase in Telomerase	Shortening of telomeres
*SFTPA1*	Modulate surface tension in the alveoli	Increased ER stress
*SFTPA2*	Modulate innate and adaptive immunity	Increased ER stress
*SFTPC*	Stabilize the surfactant fluid	Increased ER stress
*ABCA3*	Lipid transport across membranes	Increased ER stress and apoptosis
*MUC5B*	Mucin 5B production	Muco-ciliary disfunction
*TOLLIP*	Inhibitory adaptor protein within TLR	Decreased protection against ROS

^1^ *TERT*: telomerase reverse transcriptase; *TERC*: telomerase RNA component; *PARN*: poly(A)-specific RNase; *RTEL1*: regulator of telomere elongation helicase-1; *SFTPA1*: surfactant protein A1; *SFTPA2*: surfactant protein A2; *SFTPC*: surfactant protein C; *ABCA3*: ATP-binding cassette subfamily A member 3; *MUC5B*: mucin 5B; *TOLLIP*: Toll-interacting protein; TLR: Toll-like receptors; ER: endoplasmic reticulum; ROS: reactive oxygen species.

## Data Availability

Not applicable.
